# The In Vitro Toxicity Profile of Nanoencapsulated Curcumin in a Chitosan–Alginate Gel Carrier Using Rabbit Lymphocytes: Preliminary Research

**DOI:** 10.3390/ijms26178577

**Published:** 2025-09-03

**Authors:** Marta Kuchta-Gładysz, Joanna Wojciechowska-Puchałka, Anna Grzesiakowska-Dul, Michał Kmiecik, Karen Khachatryan, Gohar Khachatryan

**Affiliations:** 1 Department of Animal Reproduction, Anatomy and Genomics, Faculty of Animal Sciences, University of Agriculture in Kraków, 24/28 Mickiewicza Ave, 30-059 Cracow, Poland; marta.kuchta-gladysz@urk.edu.pl (M.K.-G.); joanna.wojciechowska-puchalka@urk.edu.pl (J.W.-P.); anna.grzesiakowska@urk.edu.pl (A.G.-D.); 2 Department of Animal Genetics, Breeding and Ethology, Faculty of Animal Sciences, University of Agriculture in Kraków, 24/28 Mickiewicza Ave, 30-059 Cracow, Poland; michal.kmiecik@urk.edu.pl; 3 Laboratory of Nanomaterials and Nanotechnology, Faculty of Food Technology, University of Agriculture in Kraków, 24/28 Mickiewicza Ave, 30-059 Cracow, Poland; karen.khachatryan@urk.edu.pl; 4 Department of Food Analysis and Evaluation of Food Quality, Faculty of Food Technology, University of Agriculture in Kraków, 24/28 Mickiewicza Ave, 30-059 Cracow, Poland

**Keywords:** nanoencapsulated curcumin, toxicity, rabbit, comet assay, micronucleus assay

## Abstract

Curcumin is a natural bioactive compound of plant origin, characterised by a wide variety of properties that make it useful in numerous industries. Furthermore, due to its health-promoting properties, such as anti-inflammatory, antioxidant, and antimicrobial effects, it has found applications in medicine and animal husbandry. Unfortunately, curcumin has low bioavailability; its hydrophobic nature means it is poorly absorbed through the gastrointestinal tract, and it is rapidly metabolised in the liver. In recent years, research has been conducted into adding nanoencapsulated active ingredients, such as curcumin, to animal feed. This research aims to improve the bioavailability and stability of these ingredients, extend their shelf life, and enhance their absorption. These effects are expected to improve overall animal health, increase production efficiency, and enhance the quality of animal products. However, a significant challenge remains: the irreversible aggregation and chemical instability of bioactive substances due to the hydrolysis of their polymeric encapsulants, which can lead to toxic effects. This study utilised peripheral whole blood from five Blanc de Termonde rabbits. In vitro cell exposure was conducted using three distinct concentrations of nanoencapsulated curcumin (C1–C3: 10, 5.0, and 2.5 µg/mL) and a control. Cytotoxicity was determined by assessing viability using trypan blue exclusion, the comet assay, and the micronucleus assay. The results indicated that all tested concentrations of nanocurcumin significantly decreased the viability of blood cells to approximately 1–9%. In contrast, the encapsulation matrices themselves were not toxic (results were statistically significant). In the comet assay, the nanocurcumin formulations were toxic at all concentrations, and the results were statistically significant. Following exposure, the micronucleus assay revealed cell damage and a high percentage of apoptotic cells (up to 30% for Cur1 at 10 ug/mL). A significant number of binucleated cells with two micronuclei (BNCs + 2MN) were also observed, again for Cur1. In view of the considerable variation in the results from the individual tests, it is advisable to repeat the research using different matrix forms and concentrations of curcumin.

## 1. Introduction

The intensive development of modern civilization and advances in the pharmaceutical industry have facilitated the production of numerous drugs impacting human health and lifespan. Nevertheless, natural products present viable therapeutic alternatives, demonstrating comparable bioactive effects. Curcumin, utilised for centuries across Asia, exemplifies such a compound. It is the principal curcuminoid extracted from the rhizome of *Curcuma longa* L. (turmeric) [1,2].

Chemically defined as 1,7-bis(4-hydroxy-3-methoxyphenyl)-1,6-heptadien-3,5-dione, curcumin is a natural, bioactive phytochemical. Its diverse biological properties underpin its broad application across multiple industries [3]. Notably, its vivid yellow pigmentation makes it valuable as a dye in the food and textile sectors [4]. Furthermore, curcumin holds significant therapeutic potential within the pharmaceutical industry, exhibiting multiple health-promoting effects including anti-inflammatory, antioxidant, antibacterial, anticancer, antidiabetic, and antirheumatic activities. Research also indicates protective effects for internal organs such as the liver, heart, and kidneys [5]. Consequently, it has a long-standing history of use in traditional medicine for the topical treatment of slow-healing wounds and managing inflammatory and gastrointestinal ailments [6].

A major limitation to curcumin’s clinical utility, however, is its inherently low systemic bioavailability following oral administration. This results from its hydrophobic nature, poor absorption from the gastrointestinal tract, and extensive pre-systemic metabolism in the liver, which inactivates the majority of the ingested compounds before they reach systemic circulation [7]. Consequently, significant research efforts focus on developing more absorbable and efficacious curcumin formulations. A promising strategy involves nanoencapsulation [1,8]. Encapsulated curcumin exhibits enhanced biological properties, including greater antioxidant and anti-inflammatory activity compared to the free compound. Crucially, encapsulation improves curcumin’s bioavailability, stability, absorption, and targeted delivery, thereby amplifying its therapeutic potential [9].

The application of biomaterials and nanotechnology has become increasingly essential in medicine, veterinary science, and the pharmaceutical and food industries to enhance the quality of life for humans and animals. Nanomaterials derived from natural polymers are of particular interest due to their versatile physicochemical properties and promising biological applicability. Chitosan and alginate are two such key natural polymers. Chitin, the second most abundant natural polymer after cellulose, is a polysaccharide found in fungal cell walls, yeast, and crustacean exoskeletons. Chitosan (Chi), a derivative obtained via partial deacetylation of chitin (typically using concentrated NaOH or enzymatic hydrolysis), possesses high application potential [10]. It demonstrates excellent biocompatibility, biodegradability, intrinsic antimicrobial activity, and low immunogenicity. As a natural fungicide and antibacterial agent, chitosan finds use in agriculture and veterinary medicine in various forms (e.g., gels, membranes, sponges, beads, and scaffolds) [11]. Its unique ability to form porous structures is exploited in tissue engineering, drug delivery systems, and wound healing applications [12,13].

Alginate (Alg), another natural polymer commonly isolated from brown seaweed [14], is also a natural polymer and it is commonly isolated from marine seaweed [14] and shares advantageous properties with chitosan, such as biocompatibility, biodegradability, non-antigenicity, and gel-forming capabilities (via ionic cross-linking). It also acts as a thickening, emulsifying, and chelating agent. A key characteristic is its good water solubility, enabling the formation of hydrogels with tunable viscosity and consistency.

This versatility grants the broad utility of alginate across pharmaceutical, food, cosmetic, paper, textile, and even petrochemical industries, formulated as gels, microspheres, foams, fibres, or sponges. Notably, the ionic interactions between chitosan (polycationic) and alginate (polyanionic) form stable polyelectrolyte complexes (PECs), enhancing the bioactivity of the composite material (Chi-Alg). This Chi-Alg composite has been successfully employed for drug/protein delivery, wound healing, and tissue engineering (e.g., tendon, ligament, and intervertebral disc) [15]. However, a persistent challenge remains: the potential for irreversible aggregation and chemical instability of encapsulated bioactive substances due to polymer hydrolysis, which could lead to toxicity.

Nanoencapsulation is a prominent nanotechnology technique. Nanocapsules, typically ranging from 10 to 1000 nanometres in size, comprise a core (containing the active substance, e.g., a drug, vitamin, or nutrient) and a protective polymeric matrix (shell). This shell, fabricated from synthetic or natural polymers, enhances shelf life, improves stability, and shields the core from degradation under unfavourable conditions. Nanoencapsulation significantly improves the bioavailability of active substances by enhancing their solubility, absorption, and ability to permeate biological barriers [16,17]. Consequently, nanocapsules find diverse applications in cosmetics, cleaning products, medicine, wound dressings, agrochemicals, food, and packaging [18,19].

The chitosan–alginate complex forms a stable system for curcumin encapsulation, which prevents its rapid release. This is achieved through electrostatic interactions between positively charged chitosan (a polycation) and negatively charged alginate (a polyanion). These interactions result in the formation of a polyelectrolyte complex that encapsulates curcumin.

The interactions between chitosan, alginate, and curcumin can significantly influence cellular toxicity profiles by modulating the compound’s bioavailability, stability, and targeted release. The polysaccharide coating (e.g., chitosan–alginate complexes) protects curcumin from degradation in the cellular environment, reducing its sudden release and potential cytotoxicity. It also slows down the release of curcumin, preventing toxic concentrations in healthy cells (e.g., liver and kidneys) [20,21,22].

The chitosan–alginate coating may enhance selectivity toward target cells. Polysaccharides modify the internalisation of curcumin primarily through endocytosis rather than passive diffusion, limiting nonspecific damage to cell membranes. Positively charged chitosan can bind to the negatively charged membranes of cancer cells, increasing local curcumin concentration in diseased tissue. Meanwhile, alginate degrades in the acidic tumour microenvironment, preferentially releasing curcumin in cancer cells [23,24].

Current research explores incorporating nanoencapsulated active ingredients into animal feed to enhance bioavailability, stability, shelf life, and absorption efficiency. This approach aims to improve animal health, increase production efficiency, and enhance the quality of animal-derived products [25]. It is, however, crucial to acknowledge that nanoencapsulation technologies may carry potential risks, including genotoxicity.

Genetic toxicology assessments frequently employ the comet assay (single-cell gel electrophoresis, SCGE) to quantify DNA fragmentation induced by genotoxic agents. This sensitive technique detects single- and double-strand DNA breaks, as well as alkali-labile sites and incomplete excision repair events [26]. The micronucleus assay, conducted in peripheral blood cells, is another well-established cytogenetic test. Micronuclei form during cell division around acentric chromosomal fragments or whole chromosomes that fail to incorporate into the main daughter nuclei. These micronuclei are smaller than the primary nucleus and provide a measure of chromosome damage. Assessing chromosomal damage is vital in genetic toxicology, as chromosome mutations represent a critical step in carcinogenesis [27].

The aim of this preliminary study, conducted in vitro, is to assess the toxicity of nanoencapsulated curcumin at different concentrations encapsulated in a chitosan–alginate matrix on rabbit peripheral blood lymphocytes, using cytogenetic assays such as comet and micronucleus and a trypan blue cell viability assessment test. The results obtained will allow the selection of suitable non-toxic concentrations of this compound in the form of nanocapsules. The form of curcumin prepared and tested in this way will be used for further stages of comprehensive research on an in vivo model, the idea being to use curcumin in animal husbandry to improve their immunity and health using natural bioactive compounds.

## 2. Results

To assess the properties of nanoencapsulated curcumin within a chitosan–alginate gel matrix and to investigate its potential genotoxic effects, samples of peripheral blood lymphocytes from five Blanc de Termonde rabbits were analysed. The cells were exposed to three different concentrations of nanoencapsulated curcumin and three different dilutions of the chitosan–alginate matrix (gel) alone. For each animal, cell viability was evaluated using the trypan blue exclusion test, the alkaline comet assay, and the micronucleus assay.

**Figure 1:**
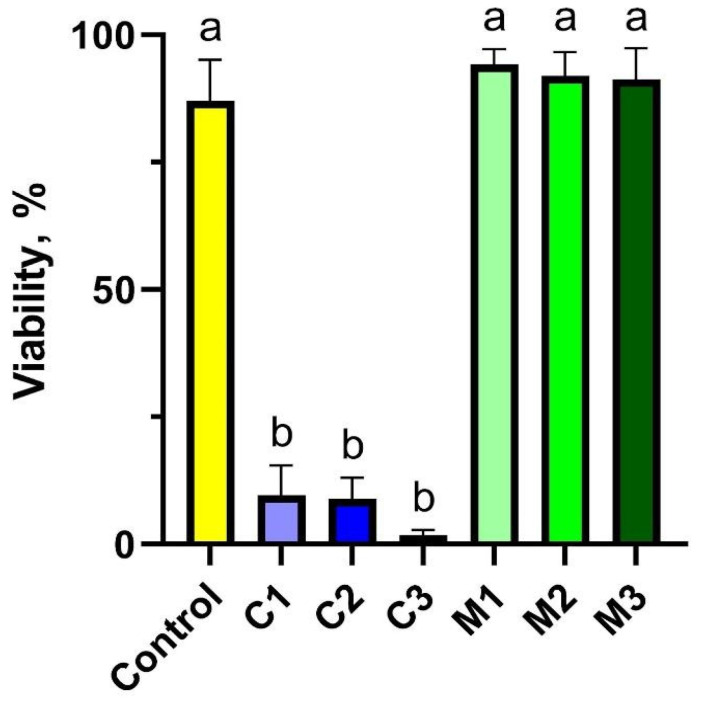
Cytotoxicity assessment of peripheral blood lymphocytes using trypan blue to quantify cell viability after exposure to chitosan–alginate gel and nanoencapsulated curcumin in the chitosan–alginate carrier: control—control group; C1—group receiving chitosan–alginate gel with nanocurcumin at a dose of 10 mg; C2—group receiving chitosan–alginate gel with nanocurcumin at a dose of 5 mg; C3—group receiving chitosan–alginate gel with nanocurcumin at a dose of 2.5 mg; M1—group receiving chitosan–alginate gel at a dose of 500 g; M2—group receiving chitosan–alginate gel at a dose of 250 g; M3—group receiving chitosan–alginate gel at a dose of 125 g. Data are expressed as the mean and standard error of the mean (SE) (*n* = 5 in each group). Values marked with different letters are significantly different at *p* < 0.05.

The experiment assessed peripheral blood lymphocyte viability across seven different experimental groups for each animal. The results indicated that the groups treated with nanoencapsulated curcumin (groups C1–C3) exhibited a toxic effect, significantly reducing cell viability to 9.60% (C1), 8.87% (C2), and 1.75% (C3), respectively. This was in contrast to the control group (C) and the groups treated with the chitosan–alginate gel alone (M1–M3), where viability remained high at approximately 90%. Interestingly, the highest concentration of nanoencapsulated curcumin (C1) demonstrated the most pronounced cytotoxic effect, resulting in the lowest viability. Detailed data are presented in Figure 1.

In the study groups following exposure to the chitosan–alginate matrix at three concentrations (groups M1, M2, and M3), there was a significant increase in viability, reaching 90% of the control level. It is evident from the results obtained that gels containing nanoencapsulated curcumin (experimental groups C1–C3) have an adverse effect on the viability of peripheral blood cells in rabbits.

**Figure 2:**
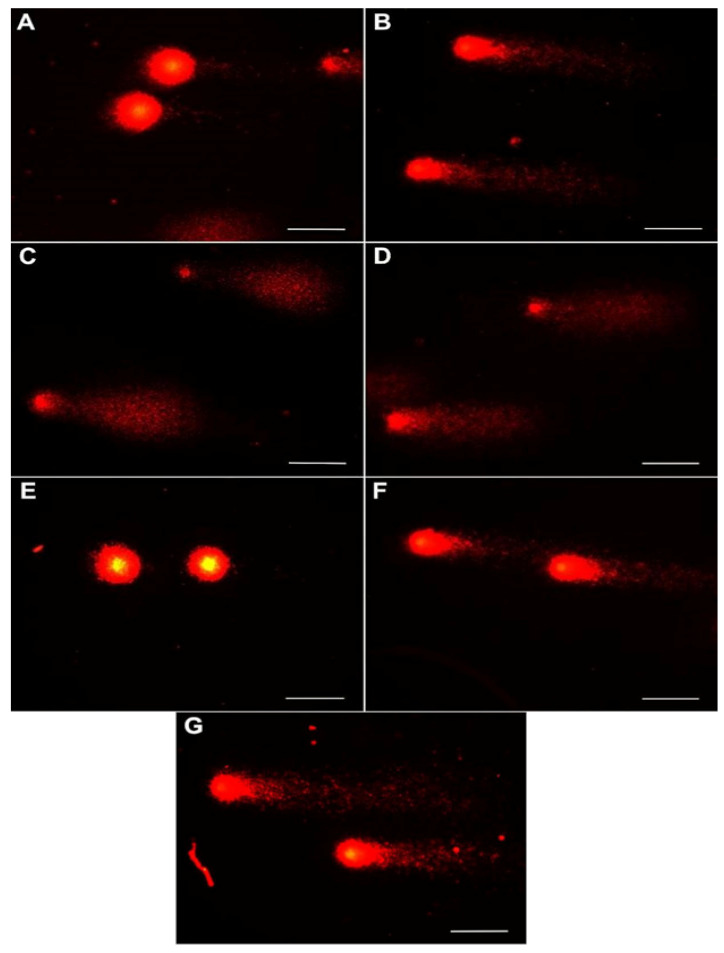
Representative images of peripheral blood cells exposed to chitosan–alginate gel and nanoencapsulated curcumin in a chitosan–alginate carrier in the comet assay: (**A**) cells from control; (**B**) cells exposed on chitosan–alginate gel with nanocurcumin at a dose of 10 mg; (**C**) cells exposed on chitosan–alginate gel with nanocurcumin at a dose of 5 mg; (**D**) cells exposed on chitosan–alginate gel with nanocurcumin at a dose of 2.5 mg; (**E**) cells exposed on chitosan–alginate gel at a dose of 500 g; (**F**) cells exposed on chitosan–alginate gel at a dose of 250 g; and (**G**) cells exposed on chitosan–alginate gel at a dose of 125 g. All scale bars show 50 µm.

**Figure 3:**
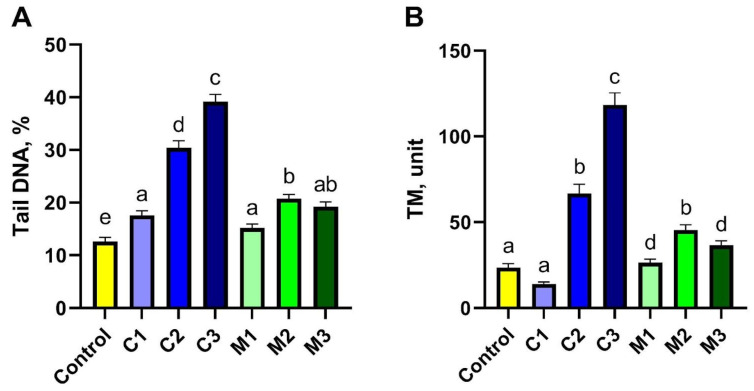
Genotoxicity assessment of peripheral blood lymphocytes using the comet assay for tail DNA (**A**) and TM (**B**) parameters after exposure to chitosan–alginate gel and nanoencapsulated curcumin in a chitosan–alginate carrier: control—control group; C1—group receiving chitosan–alginate gel with nanocurcumin at a dose of 10 mg; C2—group receiving chitosan–alginate gel with nanocurcumin at a dose of 5 mg; C3—group receiving chitosan–alginate gel with nanocurcumin at a dose of 2.5 mg; M1—group receiving chitosan–alginate gel at a dose of 500 g; M2—group receiving chitosan–alginate gel at a dose of 250 g; M3—group receiving chitosan–alginate gel at a dose of 125 g. Data are expressed as the mean and standard error of the mean (SE) (*n* = 5 in each group). Values marked with different letters are significantly different at *p* < 0.05.

Genotoxicity of the studied gels was assessed using the comet assay performed in the alkaline variant (Figure 2 and Figure 3). To obtain this goal, damage to 500 cells in each experimental group was analysed; in total, 3500 cells were included from the peripheral blood of rabbits. The main parameter that indicated the toxicity of the studied biocomposites (gels) on rabbit somatic cells was DNA percentage in the comet tail (% tail DNA). The values of % tail DNA obtained for the rabbit peripheral blood cells differed significantly between the experimental groups and the control group (*p* ≤ 0.05). The results of % tail DNA measurements for all groups are presented in Figure 2A. The addition of nanoencapsulated curcumin in bionanocomposites induced damage to somatic cells of the studied rabbits and reached a mean level of 17.55% tail DNA in the C1 group; 30.40% tail DNA in the C2 group; and 39.17% tail DNA in the C3 group compared to the control group. Moreover, significant differences were observed between all groups with added nanoencapsulated curcumin in bionanocomposites (C1, C2, C3), and the least amount of damage was caused by the highest dose of nanoencapsulated curcumin, C1, at a concentration of 10 mg/500 g emulsion. This indicates the blood protective activity of curcumin in extracorporeal settings in vitro. Meanwhile, the chitosan–alginate gel itself showed a different, more protective effect on the cells of rabbit peripheral blood, as the level of DNA degradation in this group was lower than in C1–C3 groups and equal to 15.9% tail DNA in the M1 group; 20.74% tail DNA in the M2 group and 19.22% tail DNA in the M3 group. However, the addition of chitosan–alginate gel at different concentrations (M1, M2, M3 groups) led to increased values of this parameter compared to the control group. Furthermore, a significantly higher % of tail DNA was found in the M2 group compared to the M1 group, while the M3 group did not differ significantly from M1 and M2. Tail moment (TM) was the second parameter assessed in the comet assay. This is a supplementary parameter without any unit, calculated as the product of % tail DNA and the tail length. The value of this parameter for the negative control was, on average, 23.60 ± 2.16, while for the experimental groups with the addition of nanoencapsulated curcumin, it was the following, respectively: 14.06 ± 1.16 in the C1 group; 66.67 ± 5.45 in the C2 group; and 118.44 ± 7.01 in the C3 group. On the other hand, for experimental groups using only chitosan–alginate gel, values of this parameter were obtained at the level of 26.41 ± 2.04 in the M1 group; 45.48 ± 2.97 in the M2 group; and 36.62 ± 2.58 in the M3 group. In the case of this parameter, statistically significant differences were observed between the control group and groups C2, C3, M1, M2, and M3, while differences between the control group and the C1 group were not statistically significant. Interestingly, within the experimental groups, with the addition of nanoencapsulated curcumin, the highest TM value was observed in the C3 group; this was significantly lower in the C2 group, and the lowest in group C1. And in experimental groups with chitosan–alginate gel, significantly higher values of TM were shown in group M2 compared to the M1 and M3 groups; the differences between the latter were not statistically significant (Figure 2B).

**Figure 4:**
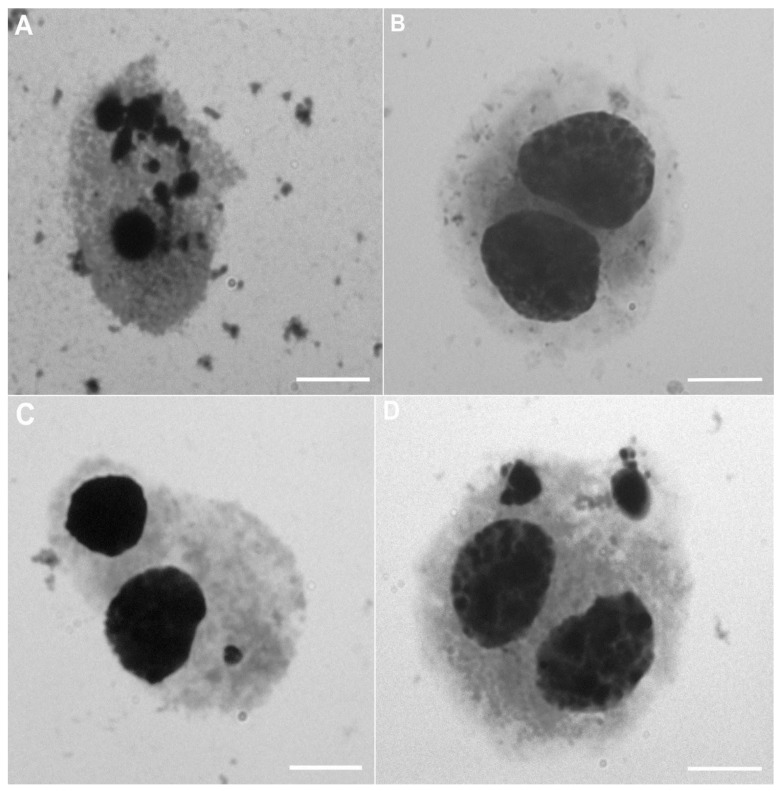
Representative images of peripheral blood cells exposed to chitosan–alginate gel and nanoencapsulated curcumin in a chitosan–alginate carrier during the micronucleus assay: (**A**) cell in apoptosis (APC); (**B**) binucleated cell (BNC); (**C**) binucleated cell with one micronucleus (BNCs + 1MN); (**D**) binucleated cell with two micronuclei (BNCs + 2MN). All scale bars show 10 µm.

**Figure 5:**
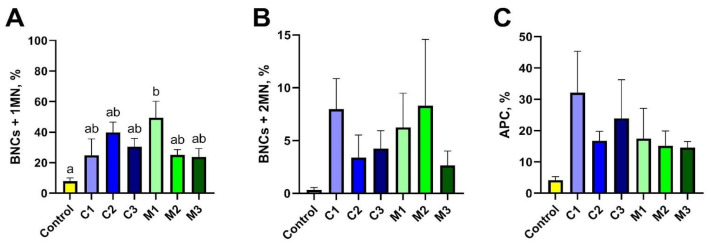
Evaluation of toxicity assessed in the micronucleus test based on BNCs + 1MN (**A**), BNCs + 2MN (**B**), and APC (**C**) parameter values after exposure to chitosan–alginate gel and nanoencapsulated curcumin in a chitosan–alginate carrier: control—control group; C1—group receiving chitosan–alginate gel with nanocurcumin at a dose of 10 mg; C2—group receiving chitosan–alginate gel with nanocurcumin at a dose of 5 mg; C3—group receiving chitosan–alginate gel with nanocurcumin at a dose of 2.5 mg; M1—group receiving chitosan–alginate gel at a dose of 500 g; M2—group receiving chitosan–alginate gel at a dose of 250 g; M3—group receiving chitosan–alginate gel at a dose of 125 g. Data are expressed as the mean and standard error of the mean (SE) (*n* = 5 in each group). Values marked with different letters are significantly different at *p* < 0.05.

The second method used in the research to assess the toxicity of nanomaterials in vitro was the cytokinesis-block micronucleus test. To estimate the genotoxic effect of nanoencapsulated curcumin, analysis of 500 binuclear cells (BNCs) was performed to assess the presence of micronuclei (MN). It was observed that the studied material contained 1 MN or 2 MNs. The presence of apoptotic cells (APCs) was an additional disturbance observed among the studied cells. A graphical representation of the results is presented in Figure 4. Based on the obtained results, no statistically significant differences were found (Figure 5C); however, the highest level of APC-type damage was found in the C1 group (32.17%), while the lowest %APC was found in groups containing chitosan–alginate gel (14.62% in the M3 group, 15.13% in the M2 group, and 17.49% in the M1 group). The BNCs + 2MN parameter (cells with two micronuclei) was the second parameter indicating the amount of cell damage. Also, statistically significant differences were not observed (Figure 5B). However, the lowest number of cells (only 0.37%) was found in the control group, and the highest amount of %BNCs + 2MN was found for the M2 group (8.29%) and the C1 group (7.97%). Statistically, significant differences were noted, however, for the BNCs + 1MN parameter (Figure 5A). The largest amount of this type of damage was shown in the M1 group (49.48%), and the lowest amount was found in the control group (7.86%).

## 3. Discussion

Due to its various properties, curcumin has increasing applications around the world, with its antioxidative properties being the most important of those in an age where dangerous diseases to civilization are emerging. Unfortunately, its poor infiltration and absorption due to poor water solubility and low biological bioavailability, remains a challenge in terms of finding an appropriate formulation of preparations containing curcumin as their ingredient. To improve the bioavailability of curcumin, a number of methods have been developed to encapsulate curcumin in liposomes and nanoparticles, complex it with phospholipids, and add essential oils or synthesise structural analogues of curcumin. Aggarwal et al. [28] studied the safety of such a curcumin–essential oil biocomplex using an in vivo and in vitro model on a Wistar rat. They showed full safety at a dose of even 1000 mg/kg of body weight of the studied rats and mice over 90 days. Nevertheless, some studies also indicate the toxic effect of curcumin in living organisms. Cao et al. [29] performed a chain polymerase quantitative analysis and immunocytochemical staining with the use of 8-hydrodeoxyguanosine in human hepatoblastoma cells, HepG2. They discovered that curcumin induced DNA damage both in mitochondrial and nuclear genomes. By using doses of curcumin of 0/2.5/5.0/10/20/40 ug/mL, respectively, they found that curcumin induced dose-dependent damage in both genomes, but the most damage was observed in the mitochondrial genome. Kuttan et al. [30] found that curcumin is cytotoxic for a broad spectrum of cancer cell lines of different tissue origin. In their opinion, curcumin activity depends on the type of cell, curcumin concentration (IC50: 2–40 μg/mL), and time of treatment (exposure). The main mechanism of curcumin-induced toxicity was the induction of apoptosis. In each case, curcumin lowered the expression of anti-apoptotic Bcl-2 proteins and increased the expression of the p53 protein. Our own research assessed the cytotoxicity of curcumin nanoencapsulated in a polysaccharide matrix (chitosan–alginate gel) at concentrations of 10/5.0/2.5 mg/mL in blood cells in vitro. Increased cytotoxicity in the form of an increased percentage of apoptotic cells was observed at the highest concentration (10 mg/mL); however, this was only found for the micronuclear assay.

Nanoencapsulation is one of the most promising nanotechnological methods, which allows bioactive compounds with proven health-promoting properties to be delivered to the organisms of humans, and animals, decreasing the risk of diseases to civilisation. The most important feature is protection and, at the same time, the controlled release of bioactive compounds at the right place and time [31]. Nanocapsules, micelles, reverse micelles, or liposomes are the most common spherical nanostructures used as carriers of bioactive compounds [32]. Our own research assessed the toxicity profile of nano/microcapsules containing curcumin. Nanoencapsulation first revolutionised the food industry, from production and processing to storage and the development of innovative materials, products and applications. The main use of nanotechnology in the food industry is innovations in its macroscale features, such as texture, taste, other sensory attributes, colouring strength, processability, and stability during shelf life, which leads to the formation of many new products. Techniques and systems based on micro- and nanoencapsulation also have huge significance in the pharmaceutical industry. The most important thing is for encapsulation materials to be safe for use in the production of medicines, and that their packaging can extend the shelf life, which is currently crucial in the battle against environmental pollution and food waste. Khachatryan et al. [33] obtained micelles that contained extracts from chokeberry marc using egg yolk powder (EYP) as an emulsifier (source of lecithin) and egg white powder (EWP) as a stabiliser. The results suggested that powder with micellar structures was more stable compared to powder obtained simply by mixing without using encapsulation methods. Woszczak et al. [8] obtained innovative food packaging in the form of two types of clingfilm based on chitosan and starch with micellar nanostructures containing extracts from turmeric rhizome and hibiscus flowers. Stanisławska et al. [1] developed an innovative, flexible biopolymer cling film based on chitosan and sodium alginate that incorporated nanocapsules containing turmeric extract. It was demonstrated that the highest encapsulation efficiency was achieved using a composite of chitosan and alginate (Chit-Alg) containing 10 mg of curcumin (Cur-2) per 500 g of matrix, resulting in a capsule size of 521 nm. Moreover, the results of microbiological tests demonstrated that this film exhibited the highest level of growth inhibition against *E. coli* bacteria, as well as against *A. expansum* fungi. In the present study, the toxicity of chitosan–alginate gels containing encapsulated curcumin at concentrations of 10, 5, and 2.5 mg per 500 g of gel, respectively, was analysed.

Methods for assessing cell viability in scientific research, particularly in toxicology, are primarily based on the detection of markers of metabolic activity, cell membrane integrity, or cell proliferation and DNA synthesis. Commonly used metabolic testing techniques include the ATP test (e.g., CellTiter-Glo), which measures ATP produced by living cells, and the tetrazolium reduction test (MTT, XTT, WST-1), which converts tetrazolium salts into coloured products via cellular enzymes. Methods for testing cell membrane integrity use dyes that differentiate between living and dead cells, including AM&IP (calcein) plus IP (propidium iodide) fluorescent staining and the trypan blue exclusion test, which differentiates between dead cells based on membrane permeability. Cell viability is also assessed using proliferation and DNA synthesis tests, which include BrdU, Edu, and clonogenicity tests. In this study, a trypan blue exclusion test was performed to determine cell viability after 24 h of exposure to the test solutions and the control. Interestingly, no toxic effects of the alginate–chitosan matrix were observed at all concentrations (M1–M3), while a significant decrease in viability was observed when the matrix was combined with nanocapsulated curcumin (C1–C3). This may indicate the positive properties of the matrix in relation to blood cells, but also its negative properties as a carrier of bioactive substances, which, in this case, is curcumin. Therefore, it would be reasonable to conduct research aimed at investigating other types of carriers for nanocapsulated curcumin (e.g., alginate) in order to rule out its potential cytotoxic nature. It is worth noting that, according to the available literature, curcumin has the ability to alleviate oxidative damage, but there is a lack of research on nanocapsulated curcumin in carriers [34]. Such action would allow for the continuation of research in an in vivo model. It would be reasonable to introduce additional cell viability tests (e.g., MTT, BrdU, or EdU) into our research, which would then allow for a broader assessment of matrix–cell interactions.

The comet assay allows individual cell nuclei to be examined, which, in turn, allows DNA damage caused by factors suspected of being genotoxic to be tested. In our research, chitosan–alginate gel with nanoencapsulated curcumin, tested at three different concentrations, was the potentially genotoxic factor. The comet method is based on electrophoretic separation of nuclear DNA, which allows for the observation of DNA fragmentation. During the analysis, the studied cells are immobilised in agarose gel. Specimens prepared in such a way are then subjected to alkaline or neutral lysis, which releases DNA from the nucleus. At this stage, electrophoresis is performed. After the separation, DNA is stained, and the obtained image is analysed under a microscope. During the analysis, direct measurement of the change in electrophoretic properties of the modified nucleic acid (as observed based on the migration of DNA strands in the electric field) is performed. During electrophoresis, the studied DNA migrates towards the anode at a speed dependent on the fragmentation degree. After the comet assay, the microscopic image of a damaged cell looks like a comet, i.e., the “head” corresponds to the site where the cell was immobilised before the lysis, and the “tail” is formed by fragments of DNA strands, relaxed and released from the nuclear structures due to breaks [26]. Depending on the pH of the electrophoretic environment, the comet assay allows various forms of DNA damage to be detected and allows two types of cell death to be distinguished—necrotic vs. apoptotic (alkaline method). Under alkaline conditions, single-strand breaks and double-strand breaks in the DNA chain were detected. Neutral conditions allow for the identification of only double DNA breaks, which are characteristic of apoptosis. In the performed research, the alkaline variant of the comet assay was used. Corona-Rivera et al. [35] investigated whether excess copper in vivo causes DNA damage, as measured using the comet assay and micronuclear assay (erythrocytic variant and EMN) in the presence of 0.2% curcumin in polychromatic erythrocytes of Balb-C mice at normal (13 ppm) and high (65, 130, and 390 ppm) copper ion concentrations. The comet assay and micronuclear assay were performed 48 h after the use of chemical substances. For both methods, a statistically significant increase in damage was observed in animals exposed to 390 ppm of copper compared to the control group or curcumin group, where the damage rate was lowered due to the presence of curcumin and its genoprotective activity. The physical length of the comet tail, expressed in um, was measured. To improve the absorption and bioavailability of curcumin, Hanna and Saad [36] prepared it in the form of nanoparticles (nanocurcumin) and then investigated its anticancer properties using human laryngeal cancer cells Hep-2. Assessment of DNA damage due to apoptosis was studied using the comet assay. Although the two parameters most commonly used to analyse comets are the “tail moment” and “% tail DNA”, this study used the calculated Olive Tail Moment (OTM), considering this parameter the best to reflect DNA damage, accounting for both the migration of the genetic material and relative amount of tail DNA (OTM = tail moment × tail DNA/100). It was found that in nanocurcumin-treated Hep-2 cells, there is a significant increase in OTM (1.9 ± 0.2) over 48 h compared to the control cells (0.46 ± 0.04), *p* < 0.001. Our research assessed the influence of chitosan–alginate gel with the addition of nanoencapsulated curcumin on the cells of rabbit peripheral blood. To assess the toxicity of the nanocomposite, an alkaline variant of the comet assay was applied, and the two parameters “% tail DNA” and tail moment (TM) were used to validate the obtained results.

The micronucleus assay (MN) is commonly used to determine chromosome damage due to various factors. What is more, this test is universal, as micronuclei can be easily identified in all cells that have a cell nucleus. In the case of mammals, the test is performed using lymphocytes, since their erythrocytes do not have a nucleus. Micronuclei are defined as chromatin structures inside the cytoplasm. Their construction is similar to that of small, additional nuclei in the cell. Micronuclei are formed as a result of the lost or delayed formation of chromosomes during anaphase of the mitotic division, or from acentric chromatin fragments (i.e., parts of the chromosome not containing a centromere). Hence, formation of micronuclei is related to chromosome number aberrations (when one or more chromosomes are lost from the cell nucleus) or to structural aberrations (loss of a fragment or one or more chromosomes). Chromosomes lost during cell division or chromosome fragments form characteristic circular structures in the cytoplasm—these are the micronuclei [37]. The cytokinesis-block micronuclear test is one variant of this technique. The CBMN assay was developed to allow an MNi assessment in binuclear cells that only underwent one division, allowing for a precise measurement of breaks or loss of chromosomes without disturbances due to non-dividing cells, which cannot express MNi. The cytokinesis-block micronucleus assay (CBMN) can be used in cell lines in vitro and in cells like lymphocytes, which can be stimulated to divide ex vivo. The CBMN assay also measures other CIN (chromosomal instability) biomarkers, such as nucleoplasmic bridges (NPBs), nuclear buds (NBUDs), and apoptotic cells (As) [38]. In our research, we used this variant of the assay, and out of all biomarkers that could be measured, we measured mainly MN and A. Farhadi et al. [39] assessed the influence of nanocurcumin in the form of micelles at a dose of 160 um/mL on the chromosomal damage of peripheral blood lymphocytes using micronuclei (MN) in patients with differentiated thyroid carcinoma (DTC) after administration of a therapeutic dose of iodine (I-131) within the scope of a clinical trial. They used the MN assay to assess toxicity, and their results confirmed that administration of curcumin as nanomicelles to patients may prevent genetic damage caused by I-131 in human lymphocytes because administration of nanocurcumin decreased the frequency of MN after a week of radioactive iodine therapy in patients treated with curcumin by 32%. Jagetia [40] aimed to determine the ability of curcumin to moderate the formation of micronuclei in human peripheral blood lymphocytes (HPBLs) exposed to 0–4 Gy of γ radiation. In the experiment, curcumin at doses of 0.125, 0.25, 0.5, 1, 2, 5, 10, 20, or 50 µg/mL was administered 30 min before irradiation. The CBMN variant of the micronucleus assay was used to investigate toxicity, and based on the obtained results, it was concluded that curcumin administered at a dose of 0.5 μg/mL limited the formation of micronuclei after γ irradiation by inhibiting the formation of free radicals caused by radiation. Rawat et al. [41] studied in vitro the ability of curcumin to prevent DNA damage caused by bile acid based on the micronucleus assay and the activity of the nuclear factor-kappaB (NF-κB) in oesophagus cell lines (OE33) using real-time PCR of the extracted RNA. Based on, e.g., the MN assay, they found that treatment of OE33 cells in vitro with curcumin at a concentration of 50 μM significantly lowered DNA damage in chromosomes. In our research on the toxicity of nanoencapsulated curcumin, the MN assay was also used as the second method. Based on the obtained results, it was found that the form of chitosan–alginate gel obtained with the addition of nanocurcumin at concentrations of 10/5/2.5 ug/mL showed toxic effects and generated damage at the level of chromosomes with a high percentage of BNC + 1MN and apoptotic cells. Thus, all cytogenetic tests used in the experiment indicate that the alginate–chitosan matrix is not a suitable carrier for the bioactive substance curcumin and, in this form, it even exhibits cyto- and genotoxic properties. Therefore, further research is needed to select a suitable and non-toxic matrix so that it can be used in in vivo studies.

## 4. Materials and Methods

### 4.1. Preparation of Nanoencapsulated Curcumin

#### 4.1.1. Materials

The following chemical reagents were utilised for the synthesis of the nanocomposites: chitosan (high molecular weight: 310,000–375,000 Da, degree of deacetylation >75%, from shrimp shells, Sigma-Aldrich, Saint Louis, MO, USA); sodium alginate (Sigma-Aldrich, Saint Louis, MO, USA); toluene (HPLC Plus, ≥99.9%, Sigma-Aldrich, Saint Louis, MO, USA); acetic acid (99.5%, Chempur, Piekary Śląskie, Poland); and deionised water, extra virgin olive oil, turmeric, and ethanol (Chempur, Piekary Śląskie, Poland 96% p.a. grade).

#### 4.1.2. Preparation of Gels Containing Curcumin Nanocapsules

The synthesis was performed according to the methodology described by Stanisławska et al. [1]. A chitosan–alginate hydrogel matrix was first prepared by combining 2% sodium alginate gel (70 °C, 700 rpm) with a 1.5% chitosan solution in 2% acetic acid (70 °C, 700 rpm, Heidolph RZR 2020, Heidolph Instruments GmbH & Co. KG, Schwabach, Germany) at a 2:1 weight ratio (alginate–chitosan). This was achieved using a homogeniser (10 min, Polytron PT 2500 E, Kinematica AG, Malters, Switzerland). Subsequently, a curcumin-loaded emulsion was prepared for incorporation into the gel. Curcumin, previously obtained via Soxhlet extraction with ethanol for over 5 h and purified via silica gel column chromatography, was used. An emulsion was formed via ultra-sonication (40 kHz for 25 min at 2 °C, Sonopuls HD 4200, Bandelin, Berlin, Germany) of a mixture containing 10 mg of the purified curcumin, 5 mL of oil, and 5 mL of water. This emulsion was then added to 490 g of the pre-formed chitosan–alginate gel. The final mixture was homogenised for 5 min (Polytron PT 2500 E, Kinematica AG, Malters, Switzerland) to uniformly disperse the emulsion within the matrix, yielding the final nanocomposite gel (C1). A control sample (C control) was prepared by adding 10 mL of water instead of the emulsion to 490 g of the chitosan–alginate gel, followed by homogenisation for 5 min under identical conditions.

The resulting gels were characterised using a UV-Vis spectroscopy (Hitachi U-2900 spectrophotometer (Hitachi Ltd., Tokyo, Japan)) and emission spectroscopy (HITACHI F7000, Hitachi Ltd., Tokyo, Japan). The results were consistent with those reported in our previous studies [1].

#### 4.1.3. In Vitro Release Study

The in vitro release profile of curcumin from the nanocomposite gel (C1) was examined using the organic acceptor phase method. Precisely 20.0 g of C1 gel was meticulously measured and transferred into a 200 mL conical flask. In order to simulate the release environment in the hydrated state and thereby facilitate curcumin diffusion, 20.0 mL of deionised water was added. Subsequently, 40.0 mL of toluene was added to the aqueous phase with the purpose of serving as an effective acceptor phase for the released curcumin. The flask was hermetically sealed to prevent solvent evaporation. The mixture was placed on a magnetic stirrer (Heidolph RZR 2020, Heidolph Instruments GmbH & Co. KG, Schwabach, Germany) and maintained at 37 ± 0.5 °C to simulate physiological temperature. The mixture was stirred at a constant speed of 300 rpm to ensure uniform mixing of the organic phase without excessive disturbance of the interface.

At specific time intervals (0, 2, 4, 10, 15, 20, 30, 40, 60, 90, 140, 180, and 320 min), 3.0 mL portions were meticulously withdrawn from the toluene layer using a glass syringe. The UV-Vis spectrum was obtained using a Hitachi U-2900 spectrophotometer (Hitachi Ltd., Tokyo, Japan). Subsequent to the measurement, the samples were expeditiously returned to the conical flask to ensure the maintenance of a constant total volume.

**Figure 6:**
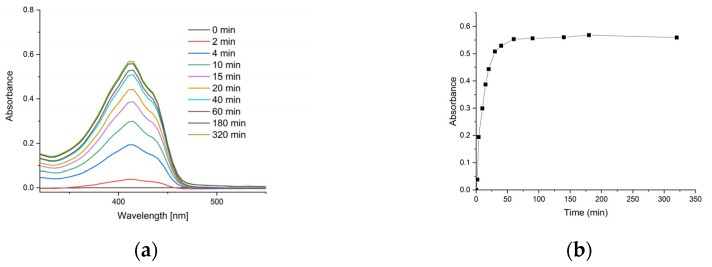
Release characteristics of curcumin from nanocomposite gel: (**a**) time-dependent UV-Vis spectra of the acceptor phase (toluene) showing increasing curcumin absorbance at λ_max_ ≈ 415 nm; (**b**) cumulative release kinetics of curcumin from chitosan–alginate nanogels.

Figure 6a presents the UV-Vis spectra of samples taken during the kinetic study, while Figure 6b shows the release kinetics of curcumin.

A kinetic analysis of curcumin’s release from chitosan–alginate nanogels into toluene was conducted. The fraction of curcumin released (M_t/M_∞) was calculated from the UV-Vis absorbance data, where M_∞ represents the maximum absorbance at equilibrium (0.569). The release kinetics were modelled using the Korsmeyer–Peppas power law model [42], defined by the equation M_t/M_∞ = k * t^n, where k is a kinetic constant and n is the release exponent indicative of the transport mechanism. The results yielded a release exponent (n) of 0.37; a kinetic constant (k) of 0.15; and a high coefficient of determination (R^2^) of 0.981. For spherical systems, an n value of approximately 0.43 is indicative of a Fickian diffusion release mechanism [43]. The obtained value of *n* = 0.37 is close to this theoretical benchmark, suggesting that the release process was primarily governed by Fickian diffusion of curcumin through the swollen polymer matrix into the acceptor phase. Furthermore, the results suggest that the complete release of curcumin was achieved within a time frame of 60 min.

### 4.2. Toxicity Profile

Study material comprised whole peripheral blood at a volume of about 6 mL, which was collected directly postmortem from 5 Blanc de Termonde rabbits in sterile sample tubes with lithium heparin (Improvacuter). Experimental animals were the same age (<3 years; 2 males and 3 females), in good health, and were kept with constant access to water and fed ad libitum, using a commercially available granulated complete feed mixture with a content of 10.2 MJ of metabolic energy, 16.5% general protein, and 14% crude fibre.

The study samples were prepared by diluting the control gel (M1) and gel containing curcumin nanocapsules (C1) twofold and fourfold with distilled water. Hence, samples denoted as M2 and M3, and C2 and C3 were obtained. The curcumin contents in samples C1, C2, and C3 were 10 mg, 5 mg, and 2.5 mg, respectively.

For each animal, the obtained isolate was divided into 7 experimental groups as follows: C-negative control, pure blood sample kept for 24 h, C1, C2, C3, M1, M2, and M3. Then, to perform 24 h of exposure, cell isolate samples were mixed with different gel variants, depending on their concentration and gel composition, and were assigned to an appropriate experimental group according to the protocol of the performed assay.

Table 1 presents the quantities of ingredients in each dilution, along with their respective designations.

#### 4.2.1. Isolation of Whole Peripheral Blood Lymphocytes

In test tubes, 4.5 mL of whole peripheral blood was mixed with 4.5 mL of Histopaque-1077 medium (Sigma Aldrich). The samples were centrifuged for 30 min at 400× *g*. The separated fraction containing lymphocytes and monocytes was transferred to a freshly prepared 10 mL PBS (Potassium Buffer Solution, Sigma Aldrich, Saint Louis, MO, USA) medium. The sample was centrifuged again for 10 min at 250× *g*. At the end of the isolation, the lymphocyte pellet was resuspended in 100 µL of PBS.

#### 4.2.2. Viability Assessment

Cell viability was assessed by staining with a 0.4% trypan blue solution. In total, 10 µL of lymphocyte isolate from the control and experimental group and 10 µL of 0.4% trypan blue (Sigma Aldrich, Saint Louis, MO, USA) were mixed on a basal slide and incubated for 2 min at room temperature. In total, 10 µL of the solution was then transferred to a chamber. Live cells (unstained) and dead cells (stained blue) were counted in three large squares under the Bürker chamber. The stain enters the dead cells due to changes in cell membrane integrity, and the cells themselves are blue in colour, while viable cells remain clear, allowing the ratio of viable cells to dead cells to be assessed in the studied material.

#### 4.2.3. Comet Assay

For each animal, a total of seven repetitions were prepared for the comet assay. In each case, 50 µL of the culture substrate RPMI-1640 (Sigma Aldrich, Saint Louis, MO, USA); 100 µL of the lymphocyte isolate; and 100 µL of the experimental gel were added to an Eppendorf tube. The process of blood cell exposure was conducted over a period of 24 h at a temperature of 20 °C.

The alkaline comet assay [44] was used to assess DNA damage. After 24 h of exposure, the 10 µL lymphocyte isolate suspended and 75 µL of LMP agarose (low melting point) (Sigma Aldrich, Saint Louis, MO, USA) were applied to basal slides coated with NMP agarose (normal melting point) (Sigma Aldrich, Saint Louis, MO, USA). Lysis of the slides was carried out for 1 h in alkaline buffer (2.5 M NaCl (Sigma Aldrich, Saint Louis, MO, USA), 0.1 M EDTANa2 (Ethylenediaminetetraacetic Acid Disodium Salt Dihydrate) (Sigma Aldrich, Saint Louis, MO, USA), 10 mM TRIS (Trizma base) (Sigma Aldrich, Saint Louis, MO, USA), and 1% Triton X-100, pH = 10 (Sigma Aldrich, Saint Louis, MO, USA) at +4 °C with limited light. Electrophoresis was conducted under alkaline conditions in a 30 mM NaOH buffer (Sigma Aldrich, Saint Louis, MO, USA), 2mM EDTANa2 (Ethylenediaminetetraacetic Acid Disodium Salt Dihydrate [diMK2]), and pH = 12.5 (Sigma Aldrich, Saint Louis, MO, USA) under limited light for 20 min at 0.6 V/cm. Neutralisation was carried out in 0.4 M Tris (Sigma Aldrich, Saint Louis, MO, USA). For detection, slides were stained with ethidium bromide at a concentration of 200 µg/mL. A lymphocyte damage assessment was performed using CASP 1.2.3b software (CaspLab, Gdańsk, Poland). For each animal, 100 comets were analysed. The parameter determining the toxicity profile in the comet assay was the percentage of DNA in the tail (% of DNA in the tail, TD %).

#### 4.2.4. Micronucleus (CBMN) Assay

The Cytokinesis-Block Micronucleus assay (CBMN) was carried out according to the method described by Słonina and Gasińska [45]. For each animal, 7 repetitions of cell culture were prepared for the CBMN assay. In each case, 4500 µL of the substrate with standard composition RPMI-1640 (Sigma Aldrich, Saint Louis, MO, USA); 250 µL of bovine serum, inactivated (Sigma Aldrich, Saint Louis, MO, USA); 50 µL Penicillin-Streptomycin antibiotic (Sigma Aldrich, Saint Louis, MO, USA); 500 µL of lymphocyte isolate suspension; and 100 µL of the experimental gel were added to the experimental Falcon tube. After 24 h of incubation at standard conditions (37.5 °C, 5% CO_2_ with constant humidity maintained), the cultures were centrifuged at 400× *g*; the supernatant was removed, and a freshly prepared culture substrate with composition identical to the initial substrate was added. In the 44th hour of lymphocyte culture, cytochalasin B at 2.5 µg/mL (Sigma Aldrich, Saint Louis, MO, USA) was added to block cytokinesis. After 72 h, all samples were centrifuged, and the supernatant was removed. Then, 5 mL of Carnoy fixative solution (a mixture of glacial acetic acid and methanol at a ratio of 1:3, Sigma Aldrich, Saint Louis, MO, USA) was added. Fixation of the cell suspension was repeated 3 times. This protocol helps recognise cells that have completed one nuclear division as binuclear (BN) cells and indicates micronuclei only in these BN cells [46]. The lymphocytes were stained with Giemsa phosphate buffer at pH 6.8 (Sigma Aldrich, Saint Louis, MO, USA). For each animal, the percentage share of binuclear (BN) lymphocytes per 1000 lymphocytes and micronuclei (MN) per 500 BN lymphocytes was evaluated. Micronuclei were identified as morphologically identical to nuclei but smaller than them and separated from them, and the percentage of BN cells with at least one micronucleus (% BNC + MN) and the number of micronuclei per BN cell (MN/BNC) were determined as the evaluation parameters.

### 4.3. Microscopic Analysis

Microscopic documentation was performed using a Zeiss Imager A2 epifluorescence microscope with AxioCam MRc5 software (ZEN 3.9, Carl Zeiss, Germany).

### 4.4. Reagents

All reagents were purchased from Sigma-Aldrich, St. Louis, MO, USA, unless stated otherwise.

### 4.5. Statistical Analysis

The results were statistically analysed using Statistica 13.0 software (TIBCO Software Inc., Palo Alto, CA, USA). The achieved results are presented as the mean and standard error (SE). To detect any significant differences between groups, a one-way analysis of variance (ANOVA) was used, and Tukey’s multiple comparisons test was performed. Before analysis, the data were tested for normality using the Shapiro–Wilk test, and homogeneity of variance was checked using the Brown–Forsythe test. For data that did not meet the assumptions of parametric tests, a non-parametric analysis was performed, including Kruskal–Wallis ANOVA with Dunn’s multiple comparisons test. Differences were considered statistically significant at *p*-values less than 0.05.

## 5. Conclusions

The results obtained lead to the conclusion that curcumin may exhibit a dual nature. The extensive DNA and chromosome damage, combined with the reduced viability, observed in rabbit lymphocytes exposed to nanoencapsulated curcumin in a chitosan–alginate gel carrier, suggest the potential cytotoxic and genotoxic effects of this compound. Further research is, therefore, required to elucidate the mechanisms underlying the adverse effects associated with this nanoencapsulated curcumin formulation. Most importantly, a non-toxic nanocapsule formulation and composition needs to be developed and optimised, and an appropriate health-promoting dose of curcumin in the nanocomposite needs to be established before the in vivo animal model testing phase.

## Figures and Tables

**Table 1:** Sample composition and curcumin concentration.

Groups	Substance (g)	Demineralised Water (g)	Tissue	Curcumin Concentration
C control	-	-	blood	-
M1	Chitosan–Alginate gel (500 g)	-	blood	-
M2	Chitosan–Alginate gel (500 g)	500	blood	-
M3	Chitosan–Alginate gel (500 g)	1000	blood	-
C1	Chitosan–Alginate gel containing 10 mg curcumin nanocapsules (500 g)	-	blood	10 mg/500 g
C2	Chitosan–Alginate gel containing 10 mg curcumin nanocapsules (500 g)	500	blood	5 mg/500 g
C3	Chitosan–Alginate gel containing 10 mg curcumin nanocapsules (500 g)	1000	blood	2.5 mg/500 g

## Data Availability

The data presented in this study are available on request from the corresponding authors.
